# The Indirect Antiglobulin (Coombs’) Test Is Specific but Less Sensitive Than the Direct Antiglobulin Test for Detecting Anti-Erythrocytic Autoantibodies and Thereby Immune-Mediated Hemolytic Anemia in Dogs

**DOI:** 10.3390/vetsci10070415

**Published:** 2023-06-26

**Authors:** Nadine Idalan, Elisabeth Müller, Urs Giger

**Affiliations:** 1 Clinic for Small Animal Internal Medicine, University of Zürich, 8057 Zürich, Switzerland; 2 Laboklin GmbH & Co KG, 4058 Basel, Switzerland; 3 Laboklin GmbH & Co KG, 97688 Bad Kissingen, Germany

**Keywords:** autoagglutination, immunodiagnostics, canine, hematology, red blood cells

## Abstract

**Simple Summary:**

In immune-mediated hemolytic anemia (IMHA), autoantibodies shorten the survival of red blood cells, thereby causing anemia. A life-threatening inflammatory and thrombotic complication may also develop in dogs with IMHA. Although pale or icteric mucous membranes and lethargy are common clinical signs, the diagnosis of IMHA is based on routine blood and immunological tests. The antiglobulin (Coombs’) test (AT) detects the presence of autoantibodies either bound to red blood cells (direct, DAT) or unbound in serum or plasma (indirect, IAT). While the DAT has been recommended for the diagnosis of canine IMHA, the value of the IAT is less clear. We tested five different IAT methods in 94 dogs suspected of having IMHA: half of which had DAT+ results. Slightly more than half of the DAT+ dogs were also IAT+ when assessed with the laboratory microtiter plate or in-clinic gel minitube kit methods. However, IAT+ results were seen in fewer than half of the DAT+ dogs when using the microcapillary, gel column, or immunochromatographic strip kit methods. Our results suggest that the DAT is superior for the diagnosis and monitoring of IMHA in dogs but that the IAT may be helpful for diagnosis when serum, but not fresh whole blood, is available from an anemic dog.

**Abstract:**

The immunodiagnostic assessment of dogs suspected of having immune-mediated hemolytic anemia (IMHA) is based on persistent autoagglutination of erythrocytes (after three saline washes), marked spherocytosis, and a positive direct antiglobulin (Coombs’) test (DAT). However, the value of using the indirect antiglobulin test (IAT) for the detection of anti-erythrocytic autoantibodies in serum from dogs suspected of having IMHA is unclear. To evaluate the IAT, leftover serum samples from a large cohort of 94 dogs suspected of having IMHA and for which DAT results were known were incubated with DAT− erythrocytes, and five IAT techniques were performed (in part with different reagents and temperatures): microtiter plate (MICRO), microcapillary, laboratory gel column, gel minitube kit (GEL KIT), and immunochromatographic strip kit. Two IAT techniques (MICRO at 37 °C and GEL KIT with rabbit anti-dog polyvalent reagent) detected autoantibodies against erythrocytes in serum from 53% and 57% of DAT+ dogs, respectively, while other IATs performed less well. Moreover, while the analytic specificity of the IAT methods compared to the DAT ranged from 96–100%, the sensitivity range was only 9–57%. Thus, we still recommend DAT for diagnosis and monitoring of IMHA in dogs but conclude that a positive IAT result may aid diagnostically when serum is available, but fresh red blood cells are not.

## 1. Introduction

The diagnosis of immune-mediated hemolytic anemia (IMHA) in dogs remains challenging and controversial in clinical practice [1,2,3]. However, an accurate diagnosis of IMHA is essential. In dogs, IMHA is often complicated by inflammation, necrosis, and thrombosis [4,5,6,7,8]. Hyperbilirubinuria (hyperbilirubinemia), reticulocytosis (polychromasia), macrocytosis, and anisocytosis are typically seen with all hemolytic anemias, not just IMHA [1,9]. There are, however, three diagnostic markers for immune-mediated erythrocytic destruction accompanying IMHA:

- Persistent autoagglutination of erythrocytes after three saline washes [2,10].
- Marked spherocytosis (≥5/high power microscopic field at × 1000 magnification) in >60% of dogs with IMHA [11], which otherwise only occurs in rare hereditary spherocytosis [12].
- A positive direct antiglobulin (Coombs’) test (DAT) [10,13].

A positive DAT result is the most direct evidence of anti-erythrocytic antibodies in dogs and other species [1,14,15]. The DAT recognizes the presence of auto- (and allo-) antibodies (and complement) on the surface of erythrocytes, while the indirect antiglobulin test (IAT) is used to detect free anti-erythrocytic antibodies in serum or plasma [16,17]. Our recent studies showed the strong clinical value of various in-clinic and laboratory DAT techniques in the diagnosis (and monitoring) of IMHA in dogs [10,13].

In contrast, the potential value of the IAT in the diagnosis of IHMA dogs [17,18] is relatively unexplored. Indeed, the use of IAT to diagnose IMHA dogs in clinical practice is quite rare [18], presumably due to the fear of interfering with alloantibodies giving false positive results [19,20,21]. However, such concerns appear unwarranted, as dogs have no clinically important alloantibodies prior to being transfused [22,23,24]. In the present study, we evaluated various IAT techniques for the detection of anti-erythrocytic autoantibodies in serum from dogs suspected of having IMHA. A comparison of IAT results with known DAT results revealed that IAT had high specificity, but low-to-moderate sensitivity compared to the DAT at 22 °C with a rabbit anti-dog IgG reagent.

## 2. Materials and Methods

### 2.1. Animals and Samples

Leftover serum samples from dogs suspected of having IMHA and for which a DAT was requested were collected at a major veterinary diagnostic laboratory (Laboklin, GmbH & Co.KG, Bad Kissingen, Germany) between January and June 2022. Samples with DAT positive (DAT+) or DAT negative (DAT−) results by a routine laboratory method were included if the leftover serum sample volume was ≥200 μL, and the serum sample was kept frozen at −20 °C until analysis (within one month). In addition, leftover ethylenediaminetetraacetic acid (EDTA)-anticoagulated blood and serum samples from dogs with normal routine blood test results (a complete blood count [CBC] and serum chemistry panel) were collected as a source of DAT− red blood cells (RBCs) to serve as negative controls. Signalment and routine blood test results (e.g., CBC and serum chemistry panel) were collected from each dog.

### 2.2. Laboratory Techniques

None of the EDTA blood samples were autoagglutinating after three saline washes based upon macro- and microscopic examinations, and thus a DAT could be performed and interpreted [10]. Leftover serum samples were incubated with washed DAT− erythrocytes, and five IAT techniques were performed (in part with different reagents and temperatures): microtiter plate (MICRO), microcapillary (CAPIL), laboratory gel column (GEL LAB), gel minitube kit (GEL KIT), and immunochromatographic strip kit (STRIP KIT) described in detail below.

#### 2.2.1. Preparation of Erythrocyte Suspensions for Indirect Antiglobulin Test

To prepare DAT− erythrocytes from control dogs, EDTA-anticoagulated blood samples, collected <1 day prior to analysis and kept refrigerated after collection, were washed three times with physiological saline, as previously described [10,13]. The DAT was performed using previously published techniques [10]. All control RBC samples tested were found to be DAT−.

For the IAT, the freshly prepared 4% non-agglutinating RBC suspension (100 μL) from control dogs was mixed with 200 μL of thawed serum samples from dogs suspected of having IMHA at the main laboratory in Bad Kissingen, Germany. Suspensions were incubated at 37 °C for 45 min to allow anti-erythrocytic autoantibodies to bind to control RBCs. After incubation, the RBC suspension was again washed three times with saline (except for the GEL KIT method), examined for autoagglutination, and then used with the different IAT techniques.

#### 2.2.2. Direct Antiglobulin Test Methods

At the routine laboratory at Bad Kissingen, Germany, the previously described microtiter plate (MICRO) DAT method [10] was used, except that a monoclonal rabbit anti-dog IgG antibody (tebu-bio, Le Perray-en-Yvelines, France) was used as antiglobulin to determine the DAT titer (dilutions up to a maximal titer of 1:2048) for each dog suspected of having IMHA. A titer of >1:4 was considered positive. Their sera were subsequently evaluated with the various IATs at the Laboklin laboratory branch in Basel, Switzerland. Furthermore, the RBC suspensions from control dogs in Basel, Switzerland, were also analyzed with the five IAT techniques to ensure that these erythrocytes were DAT− prior to incubation with serum from DAT+ and DAT− dogs.

#### 2.2.3. Indirect Antiglobulin Test Methods

Protocols for the various IAT techniques, as well as interpretation of results, were identical to those of our prior study on DAT methods in dogs suspected of having IMHA [10], except that DAT− RBCs, which were pre-incubated with serum from DAT+ and DAT− dogs, were used. Five different IAT techniques were applied with one to three antiglobulin reagents at room temperature (22 °C) and also at 4 °C and 37 °C for the laboratory MICRO IAT. All IATs were performed on all samples by one author (NI) at the Laboklin laboratory branch in Basel, Switzerland.

For the MICRO IAT method, a microtiter plate (96 wells, U-Form, Merck, Darmstadt, Germany) was used to perform an 11-step doubling dilution gradient from 1:2 to 1:2048. Three canine antiglobulin reagents were tested: IgG (tebu-bio), goat anti-dog IgG, IgM, and C3 (GAD; Canine Coombs Reagent, VMRD, Pullman, WA, USA), and rabbit anti-dog IgG, IgM, and C3 (RAD; MP Biomedicals, Solon, OH, USA). A titer of >1:4 was considered positive. The last column of the microtiter plate contained only saline and the different RBC suspensions and served as a negative control for each sample. For the gel column laboratory (GEL LAB) IAT method, neutral gel columns (NaCl Enzyme Test and Cold Agglutinin ID-card, DiaMed GmbH, Cressier, Switzerland) were used with the addition of one of three different canine antiglobulin reagents: IgG, GAD, or RAD. For the microcapillary (CAPIL) method, all three (IgG, GAD, and RAD) antiglobulins were also evaluated. The same microcapillary tube techniques as described previously were used [10]. For the immunochromatographic strip kit (STRIP KIT; Canine Labtest DAT, Alvedia, Limonest, France) and gel minitube kit (GEL KIT; Gel Test Canine DAT, Alvedia), the RAD antiglobulin (MP Biomedicals) was integrated into both test kits. The GEL LAB method was tested after washing the erythrocytes, while the GEL KIT was tested before washing. In the comparative tables, only the test results before washing were presented. For both gel tests, any reaction from 1+ to 4+ was considered positive. Double populations were considered positive (detailed in the Results section).

### 2.3. Data Analyses

The entire dataset of serum samples from DAT+ and DAT− dogs were evaluated according to their IAT results. All statistical analyses were performed with SPSS Statistics 28 software [25] and the recorded dataset (Microsoft Excel, Windows 365, Microsoft, Redmond, Washington, USA). Cohen’s and Fleiss’ kappa (κ)-values [26,27] and two-sided 95% confidence intervals (95% CI) were calculated to assess the degree of agreement with all possible pairs of IAT and DAT methods. The data were interpreted according to Landis and Koch [28], adapted by Brennan and Silman [29], as well as by a more stringent scheme by McHugh [30]. A *p*-value of ≤0.05 was considered significantly different. The normality assumption was tested with a Shapiro–Wilk test for histograms, box plots, and Q-Q diagrams [31]. If not met, a Mann–Whitney U test was performed instead of a *t*-test [32,33]. The nominal data were analyzed with chi-squared tests or, if not indicated due to the small number of samples, a Fisher’s exact test [34,35]. In addition, analytical sensitivity and specificity were calculated for each method in comparison to the MICRO DAT with anti-IgG antiglobulin at 22 °C [36].

## 3. Results

### 3.1. Signalment and Routine Blood Test Results

**Figure 1:**
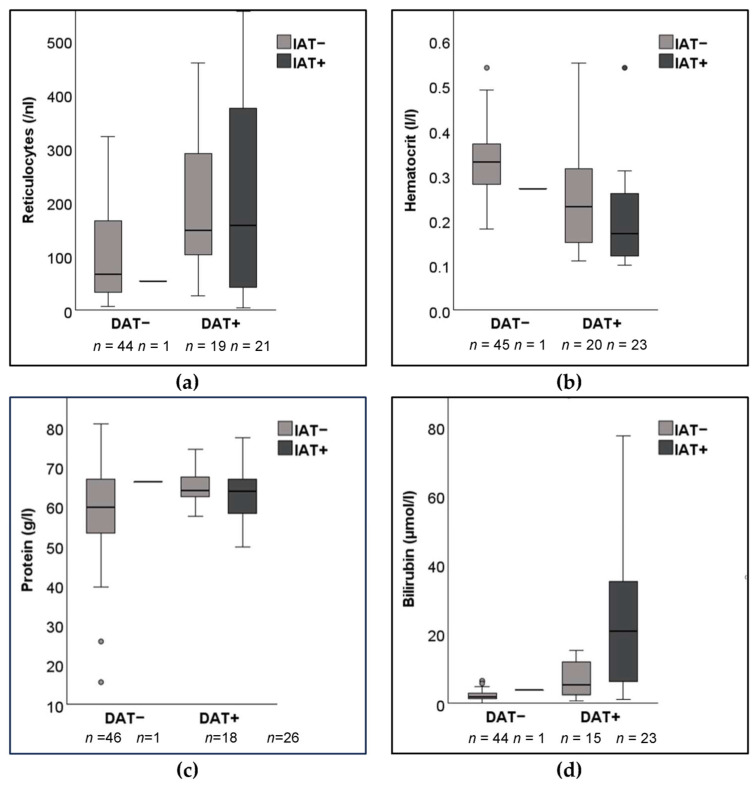
Box plots of blood parameters from 94 dogs suspected to have immune-mediated hemolytic anemia with positive (+) and/or negative (−) direct antiglobulin (DAT) and indirect antiglobulin test (IAT): (**a**) Reticulocyte count (*n* = 85); (**b**) Hematocrit (*n* = 89); (**c**) Age (*n* = 91). (**d**) Serum bilirubin concentration (*n* = 83). Data was not available for all dogs. IAT microtiter plate method with rabbit anti-dog IgG, IgM, and C3 antiglobulin at 37 °C. DAT microtiter plate method with rabbit anti-dog IgG at 22 °C.

From January to June 2022, a total of 94 leftover submitted serum samples from dogs evaluated with a DAT at a major diagnostic laboratory were included in IAT assessments. When comparing the results of the DAT microtiter plate method utilizing an IgG reagent at 22 °C, half of the dogs were either DAT+ or DAT−, respectively. Based on the signalment, there were more mixed breed dogs than purebreds overall, as well as in each group assessed (Table 1). The DAT+ dogs were significantly younger than the DAT− dogs, but there were no significant differences between IAT+ and IAT− dogs (Table 2). The DAT+ dogs were significantly more anemic and had higher reticulocyte counts and serum bilirubin concentrations than DAT− dogs, but, again, there were no significant differences between IAT+ and IAT− dogs except for the hematocrit values (Table 2 and Figure 1). Comparing the double positive (DAT+ and IAT+) and double negative (DAT− and IAT−) groups, the differences in age, hematocrit, and bilirubin values seen above (with the overall DAT+ versus DAT−) were maintained. When one only compared the IAT+ and IAT− dogs that were DAT+, no differences were observed. Because there was only one sample identified as IAT+ but DAT−, no further statistical analyses could be performed for IAT+/DAT− dogs.

While none of the EDTA-anticoagulated blood samples from the DAT+ and DAT− dogs showed autoagglutination after three saline washes, original blood smears were unavailable to microscopically determine if there was autoagglutination and/or marked spherocytosis. Results from original CBCs performed at the Laboklin laboratory did not note autoagglutination. Furthermore, insufficient data on medical history, clinical signs, and other diagnostic tests were available to determine if these dogs had primary or secondary IMHA. Thus, this study represents an analytical, diagnostic comparison of IAT methods to the routine DAT+ and DAT− results in dogs suspected of having IMHA.

### 3.2. Direct and Indirect Antiglobulin Test Results

**Figure 2:**
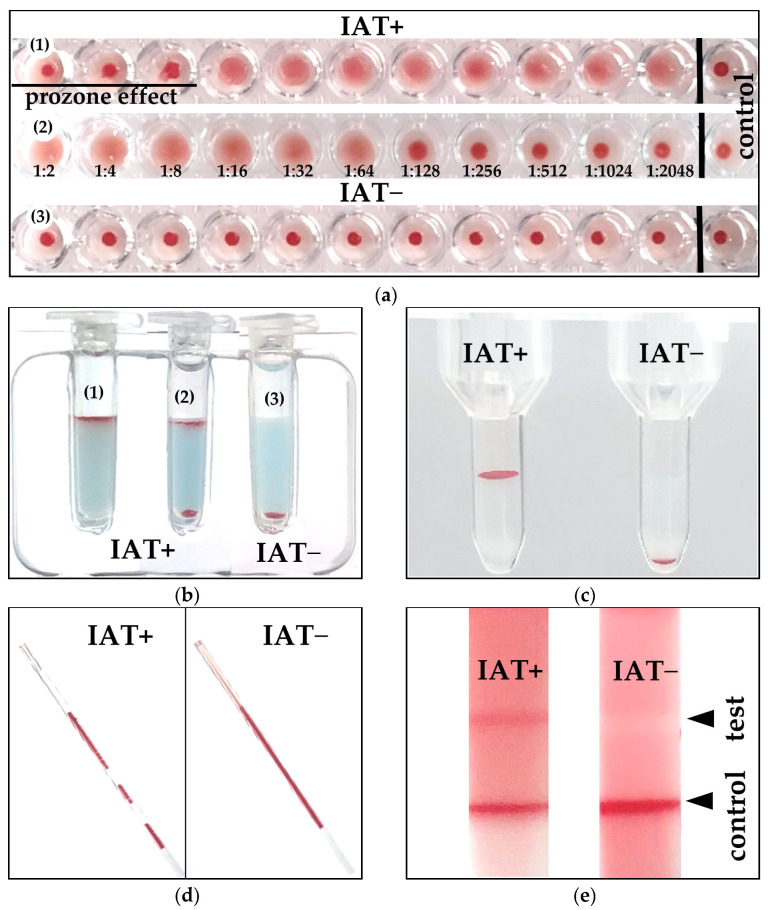
Examples of positive and negative indirect antiglobulin test results (IAT+, IAT−) obtained with different IAT methods: (**a**) Microtiter plate (**1**) 1:2048 positive with prozone effect (**2**) 1:64 positive (**3**) negative titer; (**b**) Gel minitube kit (**1**) 4+ positive reaction, (**2**) positive double population, (**3**) negative; (**c**) Gel column laboratory: 4+ and negative; (**d**) Microcapillary; (**e**) Immunochromatographic strip kit.

Using the MICRO technique with an anti-canine IgG antiglobulin at 22 °C, the DAT titer analyses clearly differentiated between moderately to strongly positive and negative DAT results (Table 3). Images of the actual IAT+ and IAT− results were very similar to those previously reported with the equivalent DAT methods [10] (Figure 2).

All serum samples from DAT dogs gave negative IAT results with all IAT techniques utilized, except those from one dog: This serum sample was from an anemic adult mixed breed dog which was IAT+ by MICRO, GEL LAB, GEL KIT, and CAPIL techniques as well as with different reagents and temperatures but was DAT−. The dog was clinically felt to have IMHA, was treated with immunosuppressive drugs, and recovered. The initial IAT results were confirmed after discovering the discordance, but the RBCs had been discarded at that time (one month later and likely would have been unusable due to hemolysis in any case). While the DAT could not be confirmed (repeated) with the original EDTA-anticoagulated blood sample, another EDTA blood sample obtained four months later from the clinically recovered dog was both IAT− (by all techniques) and DAT−, except for weakly positive MICRO and GEL KIT DAT results (Appendix A).

Among the 47 DAT+ dogs, up to 62% of the serum samples were also IAT+ with one or more IAT techniques. In a specific comparison of the MICRO DAT with IgG at 22 °C and MICRO IAT utilizing the RAD antiglobulin at 37 °C, 57% of 47 DAT+ dogs were also IAT+. Six strongly IAT+ samples showed a prozone effect with the RAD reagent.

The DAT+ and IAT+ results by MICRO technique were strong, but titers seen with the IAT were generally lower than those seen with the DAT technique (Table 3). Ignoring a one-fold antiglobulin dilution difference by MICRO techniques, when comparing DAT+ and IAT+ dogs (*n* = 27), only 4% of the samples had higher IAT titers than their respective DAT titers, 41% of the titers were equal for IAT and DAT, and 55% of the IAT titers were lower than the corresponding DAT titers. Moreover, only 24% and 36% of the DAT+ dogs were IAT+ with the RAD antiglobulin at 22 °C and 4 °C, respectively. The IAT results with IgG seemed to be temperature independent and gave similar results to RAD antiglobulin at 22 °C. In contrast to the results with the RAD antiglobulin, the GAD reagent performed poorly compared to the other IATs and the DAT, especially at 4 °C, but also at 22 °C and 37 °C (Appendix B).

**Figure 3:**
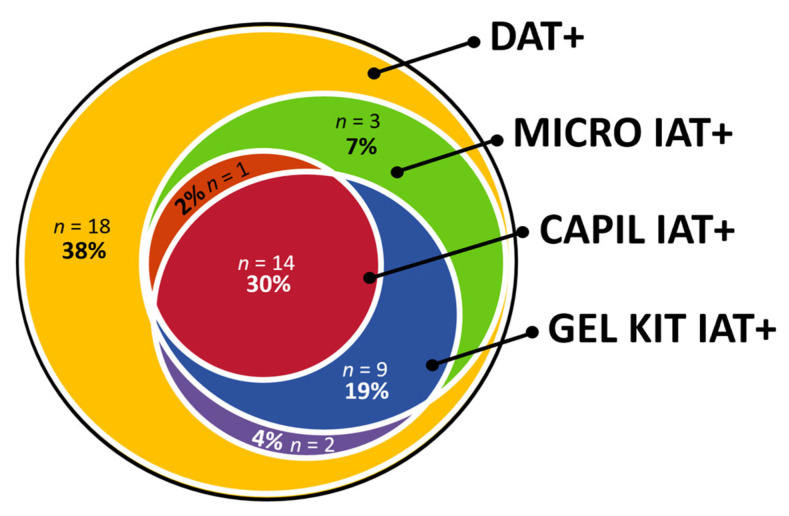
Venn diagram of three different indirect antiglobulin test (IAT) methods performed in 47 dogs suspected to have immune-mediated hemolytic anemia (IMHA) and tested positive with a direct antiglobulin test (DAT+) with a microtiter plate at 22 °C with monovalent rabbit anti-dog IgG antiglobulin. IAT methods: Microtiter plate (MICRO, 37 °C); Microcapillary (CAPIL, 22 °C); Gel minitube kit (GEL KIT, 22 °C); all tested with a polyvalent IgG, IgM, and C3 rabbit-anti-dog reagent.

Similar to the MICRO IAT with the RAD reagent performed at 37 °C, the GEL KIT IAT with the RAD reagent performed at 22 °C recognized 53% of the DAT+ dogs with mostly strong agglutination reactions (Table 4). Many IAT+ results showed a double population of cells: one IAT+ RBC population at the top of the gel of the minitube and one IAT–population at the bottom of the gel (Table 4). These results are interpreted as IAT+. Pending the assessment of kappa values, there was a good-to-very good [29] or moderate-to-strong [30] agreement between GEL KIT and MICRO IAT RAD results (Figure 3, Appendix B).

In contrast, the GEL LAB IAT with any of the three reagents at 22 °C detected only ≤17% of the DAT+ dogs. Most positive samples showed a moderately positive IAT result (2+), and one-third had a strongly positive IAT result (4+) (Table 5). In addition, only 10% of DAT+ dogs were IAT+ with the STRIP KIT (Table 6). STRIP KIT and CAPIL IAT results were non-quantitative test methods. As observed with all other methods, the GAD reagent showed the lowest sensitivity: Only 8.5% of the DAT+ dogs tested IAT+ with the CAPIL method. With the two other antiglobulin reagents, RAD and IgG, the CAPIL method detected about one-third of the DAT+ cases (32% and 30%, respectively).

### 3.3. Analytical Laboratory Specificity, Sensitivity, and Correlations

When comparing the DAT results with IgG at 22 °C to those obtained by the different IAT techniques, all IAT methods showed high analytical specificity (only one of the 47 DAT− dogs was clearly IAT+), but only poor to moderate sensitivity (Table 6).

Serum samples from DAT+ dogs were found to be IAT+ using at least one and up to five IAT techniques (Appendix C) but with different reaction strengths. The correlation of IAT+ results was moderate [29] or weak [30] (κ = 0.55) between MICRO DAT and MICRO IAT utilizing the RAD antiglobulin at 37 °C and GEL KIT IAT with RAD at 22 °C (κ = 0.51, Table 6, Figure 3). The correlation between all IATs showed predominantly a moderate [29] or weak [30] to good [29] or moderate [30] agreement with paired Cohen kappa values (Appendix B). The Fleiss′ kappa showed a moderate [29] or weak [30] agreement between all IAT methods, κ = 0.57 (95% CI, 0.55 to 0.59, *p* < 0.0001).

## 4. Discussion

The indirect antiglobulin (Coombs’) test (IAT) is routinely used in human medicine for the detection of anti-erythrocytic allo- and autoantibodies in plasma and serum to ensure blood transfusion compatibility or to diagnose IMHA, respectively [15,39]. In contrast, little is known about the value of IAT in diagnosing dogs and other domestic animal species suspected of having IMHA [17,18]. When using currently available canine antiglobulin reagents with different in-clinic and laboratory immunodiagnostic techniques to detect anti-erythrocytic autoantibodies in 94 serum samples from DAT+ and DAT− dogs, we show here that the various IAT methods are specific, but less sensitive analytical diagnostic tools to detect anti-erythrocytic autoantibodies compared to the equivalent DAT techniques reported by us recently [10,13]. Therefore, the value of performing an IAT in dogs suspected of having IMHA appears limited in comparison to the DAT but may be of value where whole blood is unavailable for analysis.

A 2019 ACVIM consensus report recognized the overall limited evidence in supporting specific methodologies for the diagnosis of IMHA in dogs but still suggested that positive autoagglutination or in-saline agglutination tests are of diagnostic value [1]. However, neither of those tests are used in human medicine, and recent studies in dogs show their lack of specificity [2,10,15]. In addition, three saline washes of EDTA-anticoagulated blood will generally remove the interference of apparent autoagglutination and allow for the best performance of DAT methods in dogs. It appears that washing of agglutinating RBCs may not be required for DAT and, shown here, IAT gel column techniques [10,13].

Numerous reports indicate that many dogs with IMHA exhibit marked spherocytosis, and the ACVIM consensus report also recommends using spherocytosis as a diagnostic criterion [1,40]. However, marked spherocytosis is also seen with hereditary spherocytosis and when examining the wrong (thick) microscopic area of a blood smear [1,12,41]. Moreover, it should be noted that a few spherocytes can be present in blood smears of many different diseases and are, therefore, not diagnostic for IMHA [1,9]. While our prior studies on DAT also carefully examined the presence of marked spherocytosis as an additional marker for IMHA, the current study focused on comparing the DAT and IAT results in dogs suspected of having IMHA [10,13].

Considering the high titer of anti-erythrocytic autoantibodies found in dogs suspected of having IMHA by the DAT methods [10,13], we hypothesized there would also be large amounts of unbound anti-erythrocytic autoantibodies in the serum of these dogs. However, we could only document free anti-erythrocytic autoantibodies in the serum of <60% of the 47 DAT+ dogs in the present study. Interestingly, while the analytic specificity was high (96–100%) in all tests performed, there were marked differences in the sensitivity of the IAT methods used compared to the DAT results reported earlier [10,13]. In this study, the most sensitive IAT methods (giving 50–60% IAT+ results in DAT+ dogs) were the MICRO IAT with RAD at 37 °C and GEL KIT at 22 °C; other IAT techniques were far less sensitive (<30%) independent of the origin of canine antiglobulin and the temperature used.

In our previous study on DAT methods, the GEL KIT method showed a double population in 17% of the GEL KIT DAT+ dogs. In contrast, this study showed that 88% of the GEL KIT IAT+ dogs revealed a double population. There is not yet a scientific explanation for this mixed reaction. As per the kit manufacturer’s recommendation, this test result was considered positive.

In contrast, human patients with IMHA generally have IAT+ results [15,42]. Interestingly, when the IAT was positive in their studies, the agglutination and binding reactions were generally moderately to strongly positive, allowing for easy differentiation between IAT+ and IAT– samples [43]. It is unclear why approximately half of the DAT+ dogs had IAT– results. It is possible that all anti-erythrocytic autoantibodies present are already bound to erythrocytes in dogs with a DAT+ and IAT– result. Alternatively, the IAT techniques used here and in other studies [19,20,21] may not have been optimized for the detection of erythrocytic autoantibodies in canine serum. Our data concur with earlier reports utilizing fewer dogs [19,20,21]. Further methodological studies may improve the sensitivity of the IAT in dogs with IMHA.

During this study, all serum samples except one from the 47 DAT− dogs were also identified as IAT−, which indicates high analytical specificity. The only dog with discordant DAT− and IAT+ results showed moderately strong IAT+ results with various methods, but when retested three months later, both the IAT and DAT results were negative or only weakly positive by all the methods also used for the IAT. Unfortunately, we could not exclude an initial mix-up of either the RBCs or serum samples from that dog or technical problems. Furthermore, based on the attending clinician and routine laboratory test results of this case, the diagnosis of IMHA remained questionable. From a clinical perspective, repeated testing of the same sample, as well as additional testing of a second sample, are recommended when clinical impression and laboratory results appear discordant. However, it should be noted that many DAT− dogs likely have non-autoimmune anemia rather than IMHA [44].

Initial attempts to establish diagnostic IAT techniques were followed by only a few additional methodological modifications and only small surveys in dogs with suspected IMHA in the 1980s (Appendix D) [19,20,21]. These IAT assays also showed high specificity but very low sensitivity. Even with enhancements such as the use of Staphylococcus protein A to detect IgG, IAT sensitivity remained low and primarily resulted in false IAT+ results [20]. A recent study in humans suggested that the use of polyethylene glycol rather than albumin improved the sensitivity of the IAT [45]. While albumin, low ionic strength saline, papain, and bromelain have been evaluated in human diagnostic laboratories, evidence that they improve sensitivity and specificity is still lacking [46]. In veterinary medicine, some early papers with poor scientific design and small numbers claim better success [21,47], but as this technique has not been universally adopted, it was likely not successful in other laboratories. These studies also suggested that IAT performed with low sensitivity and similarly high specificity compared to DAT, which is concordant with our results. Additionally, our study included a larger number of DAT+ dogs and included several laboratory IAT methods as well as in-clinic IAT kits, different reagents, and incubation temperatures. However, supplementary studies are still required to improve the detection of anti-erythrocytic antibodies in serum and/or plasma from dogs.

In human medicine, the incubation time of serum with erythrocytes is critical for obtaining a positive IAT result [48]. In the veterinary literature, reported incubation periods ranged between 30 and 60 min [19,20,21,48]. Notably, attempts to shorten the incubation time to 15 min using the enhancers mentioned above or utilizing laser incubation [49] have not yet evolved into a standard procedure. In our study, the incubation time was set to 45 min. However, as our findings indicate that sensitivity needs to be enhanced, further investigations of different incubation periods and the use of enhancers are warranted.

## 5. Conclusions

In conclusion, the MICRO IAT and GEL KIT IAT techniques appear most suited to detect anti-erythrocytic autoantibodies in the serum (and likely plasma) of dogs suspected of having IMHA. While the analytical IAT results presented here appear to be highly specific, all the methods tested were far less sensitive than the DAT. Therefore, the DAT remains the preferred immunodiagnostic tool to specifically identify anti-erythrocytic autoantibodies. However, if fresh EDTA-anticoagulated blood (refrigerated for ≤5 days) is not available, frozen serum could be used (if stored ≤ one month) in an IAT to detect anti-erythrocytic antibodies and support a diagnosis of IMHA.

## Figures and Tables

**Table 1:** Distribution of the different breeds in the positive (+) and/or negative (−) indirect antiglobulin test (IAT) and/or direct antiglobulin test (DAT) results in 94 dogs suspected to have immune-mediated hemolytic anemia (IMHA). IAT method: microtiter plate with rabbit anti-dog IgG IgM and C3 antiglobulin at 37 °C. DAT method: microtiter plate with rabbit anti-dog IgG at 22 °C.

Breed	Dogs, *n*							
	Suspect IMHA	DAT+	IAT+	DAT−	IAT−	DAT+ IAT+	DAT− IAT−	DAT+ IAT−
Mixed breed *	28	12	9	16	19	8	15	4
Labrador Retriever	5	3	2	2	3	2	2	1
Cocker Spaniel	4	2	2	2	2	2	2	0
Cavalier King Charles	3	0	0	3	3	0	3	0
Chihuahua	3	2	1	1	2	1	1	1
German Shepherd	3	0	0	3	3	0	3	0
Poodle	3	2	1	1	2	1	1	1
Appenzeller	2	2	1	0	1	1	0	1
Border Collie	2	0	0	2	2	0	2	0
Dachshund	2	2	1	0	1	1	0	1
Fox terrier	2	1	1	1	1	1	1	0
Maltese	2	2	0	0	2	0	0	2
Rottweiler	2	0	0	2	2	0	2	0
Yorkshire Terrier	2	1	1	1	1	1	1	0
Other breeds with one dog	27	16	8	11	19	8	11	8
Breed not reported	4	2	1	2	3	1	2	1
Total	94	47	28	47	66	27	46	20

* One DAT−/IAT+ mixed breed dog.

**Table 2:** Age, sex, hematocrit, reticulocytes, bilirubin, and total protein in 94 dogs suspected to have IMHA: Comparison between groups of direct antiglobulin test (DAT) and/or/nor indirect antiglobulin test (IAT) positive dogs. Data was not available for all dogs. IAT microtiter plate method with rabbit anti-dog IgG, IgM, and C3 antiglobulin at 37 °C. DAT microtiter plate method with rabbit anti-dog IgG at 22 °C.

	*p*-Values *			
Parameters	DAT+ vs. DAT−	IAT+ vs. IAT−	DAT+/IAT+ vs. DAT−/IAT−	DAT+/IAT+ vs. DAT+/IAT−
Age (*n* = 91)	**0.011**	0.090	**0.021**	0.904
Sex (*n* = 89)	0.216	0.991	0.531	0.554
Hematocrit (*n* = 89)	**<0.001**	**<0.001**	**<0.001**	0.083
Reticulocytes (*n* = 85)	**0.005**	0.123	0.11	0.789
Bilirubin (*n* = 83)	**0.009**	0.175	**<0.001**	0.055
Total Protein (*n* = 84)	**0.022**	0.096	0.053	0.789
Dogs, n	94	94	73	47

* *p* ≤ 0.05 was considered significant (shown in bold).

**Table 3:** Comparison between positive (+) and negative (−) microtiter results of direct (DAT) and indirect (IAT) antiglobulin tests in 94 dogs suspected to have immune-mediated hemolytic anemia. The DAT was performed with a rabbit anti-dog IgG antiglobulin at 22 °C; the IAT with a rabbit anti-dog IgG, IgM, and C3 antiglobulin at 37 °C.

IAT Result	IAT Titer	DAT−	DAT+					Total, *n*
		≤1:4	1:8	1:16–32	1:64–128	1:256–512	1:1024–2048	
IAT–	≤1:4	46	1	4	3	5	7	66
IAT+	1:8	0	0	1	0	0	3	4
	1:16–32	0	0	0	0	2	4	6
	1:64–128	1	0	0	1	1	3	6
	1:256–512	0	0	0	2	2	2	6
	1:1024–2048	0	0	0	0	0	6	6
Total, *n*		47	1	5	6	10	25	94

**Table 4:** Comparison of an indirect antiglobulin test (IAT) gel minitube kit method with a direct antiglobulin test (DAT) microtiter plate method in 94 dogs suspected to have immune-mediated hemolytic anemia: DAT: microtiter plate (MICRO) at 22 °C with monovalent rabbit anti-dog IgG antiglobulin and gel minitube kit (GEL KIT) at 22 °C with polyvalent IgG, IgM, and C3 rabbit-anti-dog reagent.

			MICRO IAT, *n*					
			DAT−	DAT+				
**Titer**			≤1:4	1:8	1:16–32	1:64–128	1:256–512	1:1024–2048
**GEL KIT**	IAT–	−	46	1	5	3	3	10
	IAT+	4+	0	0	0	0	0	3
		+ *	1 **	0	0	3	7	12
	Total		47	1	5	6	10	25

* Double population of cells at top and bottom of gel column. ** Only dog with DAT− and IAT+ results.

**Table 5:** Indirect antiglobulin test (IAT) results from 94 dogs suspected to have immune-mediated hemolytic anemia with the gel column laboratory (GEL LAB) method and three different antiglobulins: polyvalent IgG, IgM, and C3 goat-anti-dog (GAD); polyvalent IgG, IgM, and C3 rabbit-anti-dog (RAD); and monovalent IgG rabbit-anti-dog (IgG). Grading of test results according to gel column manufacturer from − to 4+ [37,38].

Grading	IAT GEL LAB		
	GAD	RAD	IgG
−	87	85	83
1+	1	0	2
2+	4	3	4
3+	0	1	0
4+	2	3	3
+ *	0	2	2
Total (*n*)	94	94	94

* Double population of red blood cells at top and bottom of gel column.

**Table 6:** Kappa values with interpretations as well as analytical sensitivity and specificity of five different indirect antiglobulin test (IAT) methods compared to the direct antiglobulin test method performed with microtiter plate (DAT; MICRO with IgG reagent at 22 °C) in 94 dogs suspected to have immune-mediated hemolytic anemia: Gel minitube kit (GEL KIT); Microcapillary (CAPIL); Laboratory gel column (GEL LAB); Immunochromatographic strip kit (STRIP KIT); Microtiter plate (MICRO) with different temperatures and reagents: polyvalent IgG, IgM, and C3 goat-anti-dog (GAD); polyvalent IgG, IgM, and C3 rabbit-anti-dog (RAD); and monovalent IgG rabbit-anti-dog (IgG).

IAT	Antiglobulin, Temperature, °C	Analytical Sensitivity, %	Analytical Specificity, %	Cohen’s Kappa (κ)		
				Value	Interpretation ^1^	Interpretation ^2^
GEL KIT	RAD, 22	53.2	97.9	0.51	Moderate	Weak
MICRO	RAD, 37	57.4	97.9	0.55	Moderate	Weak
	RAD, 4	51.1	97.9	0.48	Moderate	Weak
	RAD, 22	40.4	97.9	0.38	Fair	Weak
	IgG, 4, 22, 37	36.2	97.9	0.34	Fair	Minimal
	GAD, 4, 22, 37	12.8–23.4	97.9	0.10–0.21	Poor to fair	None to minimal
CAPIL	RAD, 22	31.9	95.7	0.29	Fair	Minimal
	IgG, 22	29.8	97.9	0.27	Fair	Minimal
	GAD, 22	8.5	97.9	0.06	Poor	None
STRIP KIT	RAD, 22	10.6	100	0.10	Poor	None
GEL LAB	IgG, 22	21.3	97.9	0.19	Poor	Minimal
	RAD, 22	17.0	97.9	0.14	Poor	None
	GAD, 22	12.8	97.9	0.10	Poor	None

^1^ Cohen’s kappa (κ) interpretation as per Landis and Koch [28] adapted by Brennan [29]: very good agreement (≥0.81); good agreement (≥0.61); moderate agreement (≥0.41); fair agreement (≥0.21), poor agreement (≤0.2). ^2^ Cohen’s kappa (κ) interpretation adapted by McHugh [30]: strong agreement (≥0.8); moderate agreement (≥0.6); weak agreement (≥0.4); minimal agreement (≥0.21), no agreement (≤0.2).

## Data Availability

The data presented in this study are available on request from the corresponding author [client-clinician-laboratory confidentiality].

## Appendix A. Results of Only Dog with Negative Direct Antiglobulin Test (DAT) and Positive Indirect Antiglobulin Test (IAT)

Test results of one dog with discordant anti-erythrocytic antibody results by a direct antiglobulin test (DAT with a microtiter plate at 22 °C with monovalent rabbit anti-dog IgG antiglobulin) and indirect antiglobulin test (IAT) with five different methods: Immunochromatographic strip kit (STRIP KIT); Gel minitube kit (GEL KIT); Microcapillary (CAPIL); Laboratory gel column (GEL LAB); Microtiter plate (MICRO) with different reagents: polyvalent IgG, IgM and C3 goat anti-dog (GAD); polyvalent IgG, IgM and C3 rabbit anti-dog (RAD); and monovalent IgG rabbit anti-dog (IgG).

**Day**	**Test**	**STRIP KIT**	**GEL KIT**	**CAPIL**			**GEL LAB**			**MICRO ***			**Routine Blood Tests**			
		RAD	RAD	GAD	RAD	IgG	GAD	RAD	IgG	GAD	RAD	IgG	Hct	Retic	Bili	Protein
1	IAT	**−**	**+ ****	**+**	**+**	**+**	**1+**	**+ ****	**+ ****	**1:16–32**	**1:64**	**1:256**	**27**	54.2	**3.7**	66.2
	DAT	ND	ND	ND	ND	ND	ND	ND	ND	ND	ND	**−**				
111	IAT	**−**	**−**	**−**	**−**	**−**	**−**	−	**−**	**−**	**−**	**−**	**41**	38.8	1.4	64.8
	DAT	**−**	**+ ****	**−**	**−**	**−**	**−**	**−**	**−**	**−**	**1:8–16**	**−**				
ND: Not determined; Hct: hematocrit (%); Retic: reticulocyte count (/nL, reference range <110.0); Bili: bilirubin (μmol/L; reference rage <3.4), Protein (g/L, reference range 54–75). Bold marked test results are lying outside of the norm range. * at 4 °C, 22 °C, and 37 °C; ** double population.																

## Appendix B. Comparison of Cohen’s Kappa (κ) Values of Five Indirect Antiglobulin Test (IAT) Results from 94 Dogs Suspected to Have Immune-Mediated Hemolytic Anemia

**IAT Method**	Antiglobulin	**CAPIL**			**GEL LAB**			**GEL KIT**	**STRIP KIT**	**MICRO**							
	Temperature	IgG 22 °C	RAD 22 °C	GAD 22 °C	IgG 22 °C	RAD 22 °C	GAD 22 °C	RAD 22 °C	RAD 22 °C	IgG 4 °C	IgG 37 °C	IgG 22 °C	RAD 4 °C	RAD 37 °C	RAD 22 °C	GAD 4 °C	GAD 37 °C
**MICRO**	GAD 22 °C	**0.61**	**0.66**	**0.42**	**0.45**	**0.62**	**0.59**	**0.55**	**0.42**	**0.68**	**0.68**	**0.68**	**0.57**	**0.51**	**0.70**	**0.71**	**0.84**
	GAD 37 °C	**0.62**	**0.59**	**0.54**	**0.44**	**0.75**	**0.59**	**0.43**	**0.38**	**0.53**	**0.53**	**0.53**	**0.45**	**0.39**	**0.56**	**0.72**	
	GAD 4 °C	**0.39**	**0.56**	**0.46**	**0.26**	**0.59**	**0.53**	**0.34**	**0.28**	**0.41**	**0.41**	**0.41**	**0.36**	**0.31**	**0.45**		
	RAD 22 °C	**0.68**	**0.65**	**0.34**	**0.58**	**0.56**	**0.45**	**0.71**	**0.34**	**0.86**	**0.86**	**0.86**	**0.85**	**0.77**			
	RAD 37 °C	**0.61**	**0.65**	**0.23**	**0.47**	**0.39**	**0.31**	**0.84**	**0.23**	**0.71**	**0.71**	**0.71**	**0.92**				
	RAD 4 °C	**0.56**	**0.60**	**0.26**	**0.53**	**0.45**	**0.36**	**0.86**	**0.26**	**0.79**	**0.79**	**0.79**					
	IgG 22 °C	**0.67**	**0.71**	**0.38**	**0.63**	**0.61**	**0.50**	**0.76**	**0.38**	**1.0**	**1.0**						
	IgG 37 °C	**0.67**	**0.71**	**0.38**	**0.63**	**0.61**	**0.50**	**0.76**	**0.38**	**1.0**							
	IgG 4 °C	**0.67**	**0.71**	**0.38**	**0.63**	**0.61**	**0.50**	**0.76**	**0.38**								
**STRIP KIT**	RAD 22 °C	**0.23**	**0.11**	**0.15**	**0.19**	**0.23**	**0.28**	**0.25**									
**GEL KIT**	RAD 22 °C	**0.60**	**0.63**	**0.25**	**0.51**	**0.43**	**0.34**										
**GEL LAB**	GAD 22 °C	**0.39**	**0.46**	**0.64**	**0.63**	**0.72**											
	RAD 22 °C	**0.62**	**0.68**	**0.69**	**0.66**												
	IgG 22 °C	**0.55**	**0.44**	**0.46**													
**CAPIL**	GAD 22 °C	**0.45**	**0.43**														
	RAD 22 °C	**0.80**															
IAT methods: STRIP KIT: in-clinic immunochromatographic strip kit; CAPIL: microcapillary tube; MICRO: microtiter plate; GEL KIT: in-clinic gel minitube kit; GEL LAB: laboratory neutral gel column card. Antiglobulin reagents: GAD: goat anti-dog IgG, IgM, and C3; RAD: rabbit anti-dog IgG, IgM, and C3; IgG: rabbit anti-dog IgG. Groups were compared by Cohen’s kappa (κ). Interpretation of the κ value as per Landis and Koch [28], adapted by Brennan and Silman [29]: Green: very good agreement (≥0.81); orange: good agreement (≥0.61); blue: moderate agreement (≥0.41); purple: fair agreement (≥0.21), red: poor agreement (≤0.20). Interpretation of the κ value, as per Landis and Koch [28] adapted by McHugh [30]: strong agreement (≥0.8); moderate agreement (≥0.6); weak agreement (≥0.4); minimal agreement (≥0.21), no agreement (≤0.2).																	

## Appendix C. Times a Serum Sample Has Tested Positive with Different Indirect Antiglobulin Test (IAT) Methods

Number of times a serum sample has tested positive with different indirect antiglobulin test (IAT) methods, ranging between IAT− (the sample tested negative in all five IAT methods) to 5 IAT+ (the sample was positive with all five IAT methods). Separated per IAT method. All dogs (*n* = 94) were suspected to have immune-mediated hemolytic anemia.

**IAT Method**		**Number of IAT+ Results**				
	**IAT−**	**1 IAT+**	**2 IAT+**	**3 IAT+**	**4 IAT+**	**5 IAT+**
MICRO, 37 °C	64	3	7	9	7	2
GEL KIT, 22 °C	64	2	6	9	7	2
GEL LAB, 22 °C	64	0	0	0	7	2
STRIP KIT, 22 °C	64	0	0	3	0	2
CAPIL, 22 °C	64	0	1	6	7	2
Total	64	5	7	9	7	2
IAT methods: MICRO: microtiter plate; GEL KIT: gel minitube kit; GEL LAB: laboratory gel column; STRIP KIT: immunochromatographic strip kit; CAPIL: microcapillary technique. Antiglobulin used: polyvalent rabbit-anti-dog IgG, IgM, and C3.						

## Appendix D. Comparison of Direct and Indirect Antiglobulin Test (DAT and IAT) Results in Dogs in Previously Published Studies, and the Study Reported Here

**Year of** **Publication**	**Quimby** **(1980)**	**Kaplan** **(1983)**	**Jones** **(1986)**	**Idalan** **(2023)**
Dogs studied, n	193	18	23	94
DAT+, n	15	9	8	47
IAT+, n	6	3 (6 *)	0 (23 **)	28
IAT−, n	187	15	23 (0 **)	66
Antiglobulin	poly	poly and mono IgG, IgM, and C	poly and mono IgG, IgM, and C	poly
Method (titer)	TUBES	MICRO	TUBES	MICRO
Sensitivity, %	40	33	0	57
Specificity, %	100	100	100	98
* Staphylococcus protein A enhanced for IgG IAT+; ** papain enhanced IAT+; MICRO: microtiter plate; mono: monovalent; poly: polyvalent; TUBES: tubes for each reagent concentration.				
